# Strengthening Routine Immunization Services in an Angolan *Comuna*: The Fight against the Burden of Unvaccinated Children in the Sustainable Development Goals Era

**DOI:** 10.3390/ijerph16224572

**Published:** 2019-11-19

**Authors:** Mattia Fattorini, Calistus Wilunda, Gloria Raguzzoni, Cecilia Quercioli, Gabriele Messina, Maria Pia Fantini, Giovanni Putoto

**Affiliations:** 1 Post Graduate School of Public Health, Department of Molecular and Developmental Medicine, University of Siena, Via Aldo Moro 2, 53100 Siena, Italy; 2 African Population and Health Research Center, Manga Close, Off Kirawa Road, P.O. Box 10787-00100, Nairobi, Kenya; 3 Post Graduate School of Public Health, Department of Biomedical and Neuromotor Sciences, University of Bologna, Via San Giacomo 12, 40126 Bologna, Italy; 4 Healthcare Management, Campostaggia Hospital, Local Health Unit Tuscany Southeast-Siena, 53100 Siena, Italy; 5 Department of Molecular and Developmental Medicine, University of Siena, Via Aldo Moro 2, 53100 Siena, Italy; 6 Department of Biomedical and Neuromotor Sciences, University of Bologna, Via San Giacomo 12, 40126 Bologna, Italy; 7 Operational Research Unit, Doctors with Africa CUAMM, Via S. Francesco 126, 35121 Padua, Italy

**Keywords:** vaccination, vaccine preventable diseases, routine immunization services, project evaluation, Angola

## Abstract

In May 2018, the non-governmental organization (NGO) *Doctors with Africa CUAMM* began to implement an intervention to strengthen Chiulo Hospital’s public health section to deliver immunization services in Mucope *Comuna*, Ombadja District. We aimed to evaluate the effect of this intervention. During the intervention period, actions such as staff training, improvement in the monitoring of vaccine stockpile, and the involvement of Community Health Workers were performed. The effects of the intervention on the number of vaccine doses administered were examined using negative binomial regression. Doses administered were 14,221 during the intervention period and 11,276 in the pre-intervention one. The number of administered doses was 26% higher (95% CI 9%–45%) in the intervention period than in the pre-intervention period. This was driven by vaccine doses administered during outreach sessions, where a statistically significant increase of 62% (95% CI 28%–107%) was observed. Regarding individual vaccines, statistically significant increases in the number of doses were observed for OPV2 (76%), OPV3 (100%), Penta3 (53%), PCV3 (53%), and Rota2 (43%). The NGO interventions led to improved delivery of immunization services in the study area. Greater increases were observed for vaccine doses that are more likely to be missed by children.

## 1. Introduction

Immunization represents one of the most important tools to contain global mortality: It has been estimated that childhood vaccination for 10 diseases in 41 of the poorest countries could prevent 36 million deaths between 2016 and 2030 [1]. Vaccines also show positive economic implications, with a US$44 return in economic and social benefits for every dollar spent in childhood immunization [2].

In 2012, 194 countries endorsed the *“Global Vaccine Action Plan”*, a roadmap that aims to achieve a >90% national coverage for all the vaccines in the schedule of each country by 2020 [3]. Moreover, the need to improve the control of vaccine-preventable diseases was reaffirmed by Members States of African WHO Region in the *“Regional Strategic Plan for Immunization 2014–2020”*, which defines interventions strategies in particular for the sub-Saharan context, where many countries still exhibit gaps and deficiencies in the organization of immunization programs [4].

In the literature, several interventions have been proposed to improve routine immunization services in order to increase the number of vaccine doses administered and, consequently, to obtain higher immunization coverage [5,6]. Among these, providing information to caregivers about immunization and implementing of vaccination sessions regularly in hard-to-reach communities showed positive effects, in particular when these interventions are evaluated and tailored to the context and the needs at the regional, national, and local levels, as recommended by WHO [3,4,6].

Globally, since 1974, DTP3 (third dose of diphtheria-tetanus toxoids-pertussis containing vaccine) coverage increased from <5% to 86% [7]. Despite this remarkable progress, in 2018, WHO estimated that globally, 19.4 million children did not receive DTP3 in the first year of life, of which 11.7 million (60%) lived in 10 countries. Among these countries, Angola hosted 484,000 children not receiving DTP3 in 2018, with an estimated DTP3 coverage of 59% [8].

After the end of the civil war in 2002, Angola showed sustained economic growth. However, since 2014, a prolonged diminution of oil prices led to an economic crisis with growing inflation and a reduction of expenditure in social and health sectors, particularly affecting children and the most vulnerable people [9].

*Doctors with Africa-CUAMM*, hereafter referred to as *CUAMM*, is an Italian Non-Governmental Organization (NGO) that has been supporting health service management and delivery in Angola since 2000. From 2004, the NGO has been involved in strengthening health services delivery in the Ombadja District; in particular, *CUAMM* has been collaborating with the Catholic Mission Hospital of Chiulo, a village situated in the *Comuna* (i.e., the third Angolan administrative level after province and district) of Mucope. In May 2018, the NGO began to implement a multifaceted intervention to strengthen the activities of the Public Health section of the hospital, whose main role is to carry out, at the local level, the tasks described in the *Programa Alargado de Vacinação (PAV),* the Angolan national routine immunization program. The intervention aimed to improve the organization and the delivery of the immunization services, focusing mainly on training, reorganization of the staff workload, and performance review.

According to PAV program and the available literature, routine immunization services are the key point of the implementation of the whole vaccination program [10,11]: Immunization sessions can be implemented at fixed sites (e.g., hospital, health centers) or at outreach sites in places with a shortage of health workers and local people rely on visits from the nearby health facility for vaccination.

This study aimed to evaluate the effect of the various actions performed during *CUAMM’s* intervention to strengthen the routine immunization services delivered in the *Comuna* of Mucope. Specifically, the study aimed to assesses the effect of the intervention on the number of vaccine doses administered.

## 2. Materials and Methods

### 2.1. Setting

The project was implemented in the *Comuna* of Mucope located in Ombadja, a district with about 350,000 inhabitants in the southern Angolan province of Cunene. In 2018, Mucope *Comuna* (one of the 5 *Comunas* in Ombadja District) had an estimated population of about 88,000 inhabitants, of whom 15,000 were children under 5 years of age. The area was served by the Catholic Mission Hospital of Chiulo, which also acted as a zonal referral hospital with a network of 41 peripheral health facilities (health centers and health posts). The hospital was a private non-profit facility, managed jointly by local diocese and the national government where vaccinations and other mother and child health services were provided free-of-charge. In 2018, Chiulo hospital (234 beds) performed 6182 antenatal visits and 1200 deliveries, of which 49 were caesareans.

### 2.2. Description of the Intervention

Routine immunization services were performed by the Public Health Staff (PHS) of Chiulo Hospital (5 skilled nurses, supervised by NGO expatriate medical doctors) during vaccination sessions conducted in the dedicated hospital outpatient clinic (fixed point, working 6 h per day from Monday to Friday) and in the territory of Mucope *Comuna,* with the execution of outreach sessions (usually twice a week, reaching about 8 different villages monthly). Communities served by outreach sessions were generally located in places more than 5 km away from the nearest vaccination point, difficult to be reached by walking. In 2018, 6 health facilities with a fixed immunization point (including Chiulo Hospital) were in the area of the *Comuna* of Mucope. Only the immunization point of Chiulo Hospital was able to carry out outreach sessions in the Mucope territory because of the availability of a vehicle and, an adequate number of staff to carry out both outreach and fixed sessions simultaneously. For all the vaccination points in the Ombadja District, vaccines, cold chain equipment, and other materials (syringes, safety boxes, etc.) were provided by the District Health Department (DHD) of Ombadja located in Xangongo, a town ≈30 km away from Chiulo.

From May 2018, various actions were implemented by the NGO supervisors in collaboration with the Ombadja DHD and the Chiulo Hospital management, in order to improve immunization services performed by Chiulo Hospital PHS:

1. Continuous training of the PHS, especially related to the data collection during the immunization sessions. Twice a month, a meeting between PHS and supervisors was held in order to address challenges faced during the implementation of routine immunization services, to clarify potential PHS doubts, to identify weaknesses in the activities carried out, and to share experiences and suggestions.
2. Revision of PHS working time, with personnel rotation between fixed and outreach activities. Outreach sessions were usually more exhausting because of long road trips and the high number of children to be vaccinated on a single day (up to 250). Personnel alternation between fixed point and outreach sessions was perceived as necessary by PHS.
3. Improving the monitoring of vaccine stockpile, with the creation of a dedicated sheet for registering, at the end of each session, the number of available doses for each single vaccine. In this way, trips to the Ombadja DHD to resupply vaccines were more accurately planned, avoiding both vaccine stockouts and the accumulation in the refrigerators of unneeded doses to avoid expiry before use.
4. Strengthening of the collaborations with local Community Health Workers (CHWs) and Traditional Birth Attendants (TBAs). These figures represent an essential resource in the scenario of Mucope *Comuna*, where most of the territory is not reached by phone or internet connection, and the proportion of non-Portuguese speakers is high. CHWs and TBAs were involved in creating connections with community leaders of Mucope villages, in the distribution of sheets containing the plan of Chiulo PHS outreach activities and in translating to the local language.
5. Shared decision with PHS, Chiulo Hospital management, Ombadja DHD and CHWs of the localities targeted by the outreach sessions. Outreach activity plans for Mucope *Comuna* were drafted every 4 months, with the selection of 6 to 8 villages usually reached at least once a month. Decisions to add, remove, or confirm communities in the plan were made on the basis of the estimated target population of the various communities, the number of vaccinated children in the villages in the 4 previous months, and the distances between Chiulo Hospital and the nearest immunization point.
6. Enhanced collaboration with Ombadja DHD, particularly in terms of sharing immunization data and health information of Mucope *Comuna* obtained during the outreach sessions performed by the Chiulo Hospital PHS.

### 2.3. Study Design

This study utilized a before-and-after intervention design based on routine immunization data. Our analysis was based on the number of vaccine doses administered by Chiulo PHS to children younger than 1 year in the study area. 

### 2.4. Data Collection 

The number of administered doses for each vaccine during fixed and outreach vaccination sessions was registered every working day by the PHS on dedicated sheets in the period of May–December 2018. Data were then entered into a Microsoft Excel^®^ database. At the end of each month, data were reviewed by Chiulo PHS and NGO supervisors. In addition to the number of vaccine doses administered during the intervention period, monthly data from January 2017 to April 2018 were extracted from the hospital records and included in the database.

### 2.5. Definition of Intervention Periods 

In order to assess the effect of the intervention on vaccination, data of the administered doses of the 2 years included in the study were divided in 2 different periods of the same duration (8 months): Pre-intervention period (from May to December 2017) and NGO intervention period (from May to December 2018).

### 2.6. Outcome Variables

Outcome variables were vaccine doses administered according to the immunization schedule as outlined in the Angolan PAV.

For children <1 year, the schedule was structured as follows:

- At birth: One dose of Oral Polio Vaccine (OPV0), one of Bacillus Calmette-Guérin (BCG) vaccine, and one of Hepatitis B (HepB_BD, Hepatitis B Birth Dose);
- At months 2, 4, and 6: One dose of OPV (OPV1-3), one of Pentavalent vaccine (Penta1-3, containing vaccine in DTP plus *Haemophilus influenzae* and Hepatitis B vaccine) and one of Pneumococcal Conjugate Vaccine (PCV1-3);
- At months 2 and 4, one dose of Rotavirus vaccine (Rota1-2);
- At month 4, one dose of Inactivated Polio Vaccine (IPV);
- At month 9, one dose of Measles-Containing Vaccine (MCV, from February 2018 combined measles/rubella vaccine was introduced in PAV) and one of Yellow Fever (YF) vaccine.

IPV was excluded from analysis because the vaccine was introduced in the routine immunization program in the whole district in January 2018.

### 2.7. Statistical Analysis 

Descriptive statistics were used to summarize data on vaccine doses administered according to the intervention time period. Because the data on the number of vaccine doses administered were over dispersed, i.e., the conditional variance was greater than the conditional mean—the effect of the intervention on the total number of vaccine doses administered was examined using negative binomial regression, with an incidence rate ratio option. The model contained only the exposure and outcome variables. Negative binomial regression was a generalization of the Poisson regression method suitable for over-dispersed count data. We also used negative binomial regression to examine the effect of the intervention on individual vaccine doses administered. We then pooled vaccine-specific effects using inverse variance-weighting fixed-effects meta-analysis. Because the observed effect of the intervention on the number of vaccine doses administered may be due to a secular trend, we conducted additional analysis by including data from a longer pre-intervention period (from January 2015 to April 2018). To make reasonable comparisons, we divided this extended pre-intervention period into 5 8-month periods (January–August 2015, September 2015–April 2016, May–December 2016, January–August 2017, and September 2017–April 2018). We then used negative binomial regression to assess changes in the number of vaccine doses administered over time with reference to January–August 2015 period. Data were analyzed using Stata^®^ version 14 (StataCorp, College Station, TX, USA) and two-sided *p* values <0.05 were considered statistically significant.

### 2.8. Ethical Considerations 

This study involved counting the number of vaccine doses and did not contain data involving human subjects.

## 3. Results

Following the guidelines of the Angolan PAV immunization schedule for children younger than one year, Chiulo PHS administered a total of 19,746 vaccine doses in 2018 (the year including the 8 months of the NGO intervention period) compared with 15,349 doses in 2017, which was an increase by 4397 (+28.7%) doses. Of these doses, 13,149 (11,090 in 2017, +18.6%) were administered at Chiulo Hospital fixed point, and 6597 (4259 in 2017, +54.9%) were administered during outreach sessions performed in the Mucope *Comuna* territory.

The number of vaccine doses administered was 14,221 during the NGO intervention period and 11,276 in the comparable pre-intervention period. Vaccine doses administered at the hospital fixed point were 8880 in the NGO intervention period and 7988 in the pre-intervention period, while the number of doses administered during the outreach sessions was 5341 in the NGO intervention period and 3288 in the pre-intervention period.

Based on negative binomial regression, the number of administered doses was 26% higher (95% CI 9–45%, *p* = 0.001) in the NGO intervention period than in the pre-intervention period. The number of doses administered during outreach session showed a statistically significant increase of 62% (95% CI 28–107%, *p* <0.001), while the increase in the number of doses administered in the NGO intervention period in the hospital was not statistically significant (11%; CI −4%–29%, *p* = 0.16).

**Figure 1:**
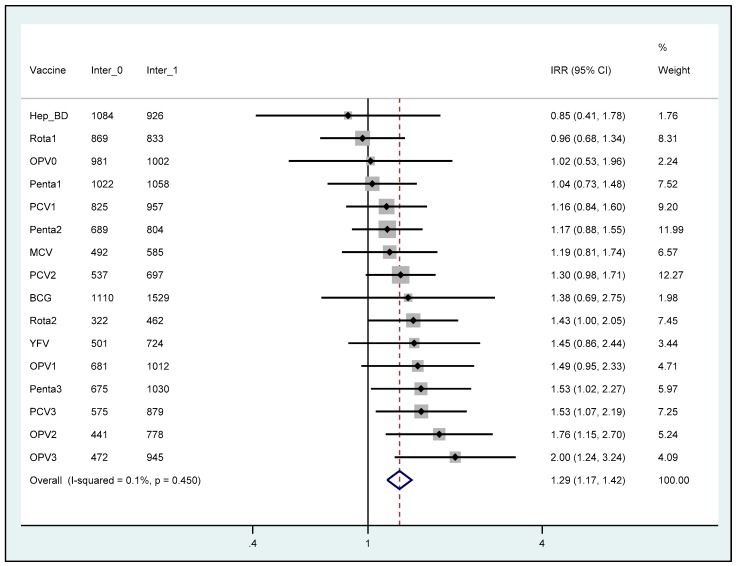
Effect of the intervention (expressed as incidence rate ratios) on individual and total vaccine doses administered by Chiulo PHS. The number of vaccine doses in the NGO intervention period (May–December 2018) is compared to the number administered in the pre-intervention period (May–December 2017). The number of vaccine doses administered in the pre-intervention and intervention periods is represented by Inter_0 and inter_1, respectively. The bars represent 95% confidence intervals around point estimates.

Regarding individual vaccines, regression analyses showed statistically significant increase in the number of doses in the NGO intervention period for OPV2 (76%; 95% CI 15%–170%, *p* = 0.009), OPV3 (100%; 95% CI 24%–224%, *p* = 0.005), Penta3 (53%; 95% CI 2%–127%, *p* = 0.037), PCV3 (53%; 95% CI 7%–119%, *p* = 0.021) and Rota2 (43%; 95% CI 1%–105%, *p* = 0.047) as shown in Figure 1. The pooled results showed a 29% (95% CI 17%–42%) higher total number of administered vaccine doses in the intervention period than in the pre-intervention period, with no evidence of heterogeneity across vaccine types.

**Figure 2:**
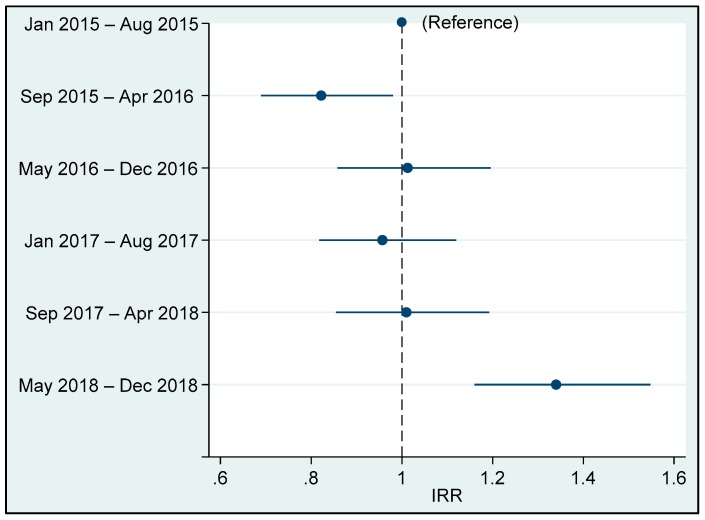
Change in the number of vaccine doses administered by Chiulo PHS from 2015 to 2018. The 48-month period was divided into 6 periods of 8 months each. The incidence rate ratios represent the relative change in the number of vaccine doses in each period with reference to the period January 2015 to August 2015. May 2018 to December 2018 represents the NGO intervention period. The bars represent 95% confidence intervals around the point estimates.

Figure 2 clearly shows an increase in the number of vaccine doses administered in the intervention period and no significant trend in the extended pre-intervention period (from January 2015 to April 2018).

## 4. Discussion

The control of vaccine-preventable diseases will continue to play a key role in the Sustainable Development Goals (SDGs) era. In order to contain the under 5 mortality rate to no more than 25/1000 live births in every country by 2030 (SDGs target number 3), actions to strengthen immunization programs will be essential [12]. These interventions are now more compelling in the WHO African Region, where the burden of unvaccinated and under-vaccinated children is almost as high as in all the other Regions combined [7].

To strengthen the immunization activities of Chiulo PHS, the NGO and the local health authorities carried out a multifaceted intervention aimed at improving the activities of the hospital and/or the outreach services. Regarding the outreach sessions, the implemented interventions were inspired by the “Reach Every District” (RED) approach, which aims to strengthen immunization programs through actions (e.g., planning and management of resources, linking health services with the health community, etc.) focused on a district level [13]. In this study, villages served by outreach sessions were located at variable distances from Chiulo Hospital (from 6 to 70 km), and the implemented actions in the NGO interventions period were mainly focused on the reduction of the burden of unvaccinated children in these communities. This approach, tailored on the complexity of the territory of Mucope (a rural *Comuna* with a shortage of fixed immunization points and a population distributed sparsely in small villages), as recently suggested by WHO, is shifting from a RED to a REC (“Reaching Every Community”) approach, and it appears to be the most effective pathway to improve the access and the quality of vaccination programs especially in rural and hard to reach areas [5,11].

Our analyses show that interventions implemented by the NGO during the project intervention period were more effective in delivering vaccine doses to children in need, especially through outreach services. These could be related to the NGO’s approach, which, with the support of CHWs and TBAs, strengthened relationships with communities in the *Comuna*. On the other hand, Chiulo Hospital registered a decrease of 800 deliveries in 2018 compared to 2017, leading to a diminution of HepB_BD administered during the NGO intervention period. According to the PAV schedule, this vaccine should be administered within the first day of the life of a child, and this can be done more easily when childbirth occurs in a hospital, rather than at home. Moreover, regression analysis highlighted a statistically significant increase in the number of administered doses in NGO intervention period for the last scheduled dose (i.e., completion of the immunization series for a single vaccine) of OPV, Penta, PCV, and Rotavirus vaccines. Thus, it is clear that the intervention contributed to the completion of the immunization schedule and hence reduction of vaccine drop-out rates. Completion of a vaccination series is essential for adequate immunity against a specific disease. During the routine training of the PHS, the project emphasized the need to inform caregivers about the benefits of vaccines and the importance of not missing future vaccinations in order to minimize the dropout rate. Differences in the number of doses of vaccines that should have been administered at the same time (e.g., Penta3, OPV3, and PCV3) were primarily related to vaccine stockouts that affected the District of Ombadja during our study period.

Outreach sessions can represent an opportunity to integrate immunization services with several other health interventions, such as deworming and vitamin A supplementation, distribution of insecticide-treated nets, screening for acute malnutrition, and antenatal care (ANC) especially in remote rural areas [5,14]. During outreach sessions, the PHS usually integrates some of these services in routine immunization activities, and during the NGO intervention period, this integration was improved in order to provide a more comprehensive public health package for the territory of Mucope *Comuna*. Vitamin A Deficiency (VAD) represents one of the most assessed micronutrient deficiencies in the world, and it is a major cause of preventable childhood blindness [15]. VAD level in children <5 years old in Angola has been defined as “severe”, with an estimated VAD prevalence of 65% [16]. For Angolan children aged less than 1 year, it has been scheduled the administration of two doses of vitamin A, at months 6 and 9. During 2017, 760 doses of vitamin A were administered by Chiulo PHS during outreach sessions, while in 2018, the number of doses increased to 807 (+5.8%) despite a stockout at the district level, which stopped vitamin A administration for 50 days in September/October. In addition, during the NGO intervention period, a skilled nurse was integrated into the PHS team to provide ANC during outreach sessions. Communities visited by immunization outreach sessions were often located far away from the nearest health facility with personnel skilled in ANC. Thus, women living in those villages are more likely to deliver at home with no ANC contacts. From July to December 2018, there were 527 ANC contacts during outreach sessions, and these were accompanied by the distribution of the recommended nutritional supplements and delivery of malaria intermittent preventive treatment in pregnancy [17]. Moreover, several women with high-risk pregnancy identified during outreach were referred to the Chiulo Maternity Waiting Home (MHW): A structure located close to the hospital where women with high-risk pregnancy are referred for close monitoring. In 2019, Chiulo PHS, collaborating with the staff of the Pediatric Unit, started to implement the delivery of extra services such as screening for malnutrition and nutritional education during outreach sessions. To be successful, the integration of these health services must be carefully planned and monitored in collaboration with key stakeholders (local health staff, NGOs, community leaders, etc.) [18,19].

Comparing district immunization experiences in three different countries (Ethiopia, Cameroon and Ghana), the adaptation of vaccination services accordingly to community needs and conditions was identified by LaFond et al. [20] as one of the four direct drivers of immunization coverage improvement; the other three were the involvement of CHWs, the promotion of partnership between health system and community, and the regular review of the performance both of the immunization program and healthcare staff. In this study, interventions performed by the collaboration of the NGO, the local health authorities, and the hospital management were all implemented following these drivers. Although the contribution of every single driver in the improvement of immunization services varies depending on each district context, they act in a synergistic way to overcome the most common barriers of good quality vaccination services in rural areas. These include lack of trained health staff and materials, transportation difficulties in reaching remote villages, vaccine stockouts, cold chain maintenance and language barriers [21].

Since 1978, the concept of Primary Health Care has been reinterpreted in different ways: Recently, 40 years after the Alma Ata Declaration, a more comprehensive definition of PHC delineate it as an approach aiming to provide health and well-being with equitable distribution according to people’s preferences and needs, in a continuum of care from health promotion and disease prevention until palliative care [22]. To achieve this vision of PHC in the 21st century, specific transformational actions in healthcare policies are required. Among these actions, WHO and UNICEF highlight the need for a different role of hospitals in the future of PHC, in order to end the dichotomy between these structures and the first levels of care ensured outside them [23]. In this vision, hospitals should move towards a people-centered organization responsible for population health jointly with other care providers, and not only focused on patients requiring acute and highly specialized treatments. Moreover, in the 21st century, hospitals should play an important role in education, promotion, and prevention, becoming prominent providers of public health services. The interventions implemented by the NGO to strengthen immunization services were geared at shifting Chiulo Hospital towards the new role of a hospital in PHC, for example the actions targeted at PHS education and the integration of different healthcare services (e.g., ANC visits, vitamin A supplementation, etc.) during vaccination sessions.

Angolan economic crisis and the consequent inflation led to a more than 50% reduction in health care expenditure in 2018 compared to 2014, despite population growth of 3.4 million in the 2014–2018 period [24]. In addition, in 2017, Angola transitioned out of the support of the Global Alliance for Vaccines and Immunization (GAVI, a public-private partnership for the increase of access to immunization services in poorest countries) [25]. The end of this support was planned before the start of the economic crisis (when Angola abandoned the status of low-income countries required to be eligible for GAVI support), which primarily affected the availability of skilled health staff. Nowadays, national immunization programs of several middle-income countries such as Angola are facing unprecedented financial problems because the end of the levels of funding and support guaranteed when classified as low-income [26]. For these reasons, Angolan PAV now requires support, especially in the delivery of immunization services and the contribution offered by the private sector could be effective in reducing the burden of unvaccinated children [27].

This study has several limitations. First, the results concerning administered doses may have been influenced by reporting bias: For example, Ombadja District borders with Namibia, and local people often access health services (including immunization) across the borders in both countries [28]. This particular situation could also lead to difficulties in the interpretation of vaccination cards due to different schedules and languages, resulting in errors in data collection regarding the number of doses administered. Second, this study did not account for other factors and secular trends that might have influenced the number of vaccine doses administered. Nonetheless, Chiulo Hospital’s immunization data from 2015 did not reveal any major change in the immunization trend in the pre-intervention period, as highlighted in Figure 2. Thus, the observed increased in immunization doses in the intervention period is unlikely to be due to a secular trend. Moreover, there was no other specific intervention to increase immunization during the study period in the Mucope *Comuna*.

## 5. Conclusions

The NGO interventions led to an improvement in the delivery of immunization services by Chiulo Hospital PHS in Mucope *Comuna*. Greater increases were observed for vaccine doses that are more likely to be missed by children. The integration of immunization services during outreach with other health services may increase the accessibility of primary health services in hard-to-reach rural areas.
